# A randomised-controlled feasibility study of the REgulate your SItting Time (RESIT) intervention for reducing sitting time in individuals with type 2 diabetes: study protocol

**DOI:** 10.1186/s40814-021-00816-0

**Published:** 2021-03-19

**Authors:** Daniel P. Bailey, Charlotte L. Edwardson, Yannis Pappas, Feng Dong, David J. Hewson, Stuart J. H. Biddle, Marsha L. Brierley, Angel M. Chater

**Affiliations:** 1 Sedentary Behaviour, Health and Disease Research Group, Brunel University London, Kingston Lane, Uxbridge, UB8 3PH UK; 2 Division of Sport, Health and Exercise Sciences, Department of Life Sciences, Brunel University London, Kingston Lane, Uxbridge, UB8 3PH UK; 3 Leicester Lifestyle and Health Research Group, Diabetes Research Centre, University of Leicester, Leicester General Hospital, Leicester, LE5 4PW UK; 4 NIHR Leicester Biomedical Research Centre, Leicester General Hospital, Leicester, LE5 4PW UK; 5 Institute for Health Research, University of Bedfordshire, Luton, LU1 3JU UK; 6 Department of Computer and Information Sciences, University of Strathclyde, G1 1XH, Glasgow, UK; 7 Centre for Health Research, University of Southern Queensland, Springfield, QLD 4300 Australia; 8 Institute for Sport and Physical Activity Research, Centre for Health, Wellbeing and Behaviour Change, University of Bedfordshire, Polhill Avenue, Bedford, MK41 9EA UK

**Keywords:** Sedentary behaviour, Prolonged sitting, Physical activity, Behaviour change, Diabetes, activPAL

## Abstract

**Background:**

People with type 2 diabetes mellitus (T2DM) generally spend a large amount of time sitting. This increases their risk of cardiovascular disease, premature mortality, diabetes-related complications and mental health problems. There is a paucity of research that has evaluated interventions aimed at reducing and breaking up sitting in people with T2DM. The primary aim of this study is to assess the feasibility of delivering and evaluating a tailored intervention to reduce and break up sitting in ambulatory adults with T2DM.

**Methods:**

This is a mixed-methods randomised controlled feasibility trial. Participants (*n*=70) with T2DM aged 18-85 years who sit ≥7 h/day and are able to ambulate independently will be randomly allocated to receive the REgulate your SItting Time (RESIT) intervention or usual care (control group) for 24 weeks. RESIT is a person-focused intervention that delivers a standardised set of behaviour change techniques to the participants, but the mode through which they are delivered can vary depending on the tools selected by each participant. The intervention includes an online education programme, health coach support, and a range of self-selected tools (smartphone apps, computer-prompt software, and wearable devices) that deliver behaviour change techniques such as self-monitoring of sitting and providing prompts to break up sitting. Measures will be taken at baseline, 12 and 24 weeks. Eligibility, recruitment, retention and data completion rates will be used to assess trial feasibility. Sitting, standing and stepping will be measured using a thigh-worn activity monitor. Cardiometabolic health, physical function, psychological well-being, sleep and musculoskeletal symptoms will also be assessed. A process evaluation will be conducted including evaluation of intervention acceptability and fidelity.

**Discussion:**

This study will identify the feasibility of delivering a tailored intervention to reduce and break up sitting in ambulatory adults with T2DM and evaluating it through a randomised controlled trial (RCT) design. The findings will inform a fully powered RCT to evaluate the effectiveness of the intervention.

**Trial registration:**

ISRCTN, ISRCTN14832389; Registered 6 August 2020.

## Introduction

There are more than 4 million people with type 2 diabetes mellitus (T2DM) in the UK, costing the National Health Service (NHS) 10% of its annual budget [1]. People with T2DM have a high risk of cardiovascular disease (CVD), early death, poor psychological well-being and a burden of diabetes-related complications, e.g. neuropathy, nephropathy and retinopathy [2, 3]. Glycaemic control is important for reducing the onset of these secondary outcomes [4, 5] making this an important target for intervention.

On average, adults with T2DM spend 8.0-9.5 h/day being sedentary (i.e. expending minimal amounts of energy when sitting, reclined or lying down during waking hours) when measured with accelerometers [6–8]. Sedentary behaviour is detrimentally associated with the duration of hyperglycaemia, waist circumference, insulin resistance and high-density lipoprotein cholesterol (HDL-C) in people with T2DM [6, 9]. The associations of sedentary time with T2DM, CVD and psychological well-being have been shown to be independent from physical activity levels [10–12], although 60-75 min/day of moderate-intensity physical activity may offer protection against the increased mortality risk associated with high amounts of sitting [13]. However, this amount of moderate activity may be an unrealistic target for the majority of the population [14]. Reducing daily sitting time is thus an important target for the prevention of CVD and poor psychological well-being in ambulatory adults with T2DM [15].

In addition to reducing daily sedentary time, regularly breaking up prolonged periods of sedentary time with short bouts of activity is recommended for people with T2DM [15]. This is largely based on findings from controlled laboratory studies in which breaking up sitting with 2-5 min of standing, light and moderate-intensity walking every 20-30 min acutely improved cardiometabolic health in healthy, overweight/obese and T2DM participants [16–19]. The magnitude of response in such studies appears to be greatest in individuals with poor metabolic health, suggesting that participants with T2DM could benefit most from this type of intervention.

A number of interventions using behaviour change techniques (BCTs) delivered through means such as education sessions, wearable devices, height-adjustable workstations and health coaching have reduced sitting in the general population and office workers [20, 21]. Multi-component workplace interventions that included providing information, goal setting, health coaching, self-monitoring and a height-adjustable workstation reduced sitting by 45-83 min per work shift after 12 months [20, 21]. A workplace intervention consisting of computer-prompt software and phone apps (for self-monitoring and goal setting), but without provision of height-adjustable workstations, reduced prolonged sitting by 39 min and increased the number of breaks from sitting by 7.8 per work shift after 8 weeks [22]. In an 8-week non-workplace specific intervention, a phone app that provided information and facilitated self-monitoring, goal setting and prompts in relation to sitting was found to be acceptable and had preliminary efficacy for increasing breaks in sitting and improving metabolic and psychological health [23]. Interventions incorporating BCTs such as providing information on health consequences, problem solving, goal setting, action planning, social support, restructuring the physical environment, prompts and cues, and self-monitoring thus appear to have promise for reducing sitting. However, there is a paucity of interventions aimed at reducing sitting in people with T2DM.

Tailored behaviour change strategies are more likely to lead to effective outcomes, as seen when delivering interventions using motivational interviewing [24, 25] and those based on self-determination theory [26]. Thus, enabling participants to select their own tools (e.g. the phone app, computer-prompt software or wearable tracker they use), whilst ensuring that the same BCT strategy is being delivered (e.g. self-monitoring), may be a more effective approach to reducing sitting. Although the findings of the interventions discussed above are promising, participants in these studies were not provided a choice with regards to the behaviour change tools they could use within the intervention. One previous intervention that comprised of reviewing sitting time, providing feedback on sitting and allowing participants to choose six different goals relating to ways to reduce sitting, led to a 96 min/day decrease in sitting after 6 weeks in older adults [27]. Interventions aimed at reducing and breaking up sitting in people with T2DM may thus benefit from using a tailored approach, but this has not yet been evaluated.

The primary aim of this study, therefore, is to conduct a randomised controlled trial (RCT) to assess the feasibility of delivery and evaluating a novel tailored intervention that utilises a standardised set of BCTs to reduce and break up sitting in ambulatory adults with T2DM. The main objectives of the study are to establish and refine a recruitment strategy, determine participant attrition and completion rates for the outcome measures, assess the acceptability of randomisation to intervention or usual care, acceptability of the intervention and data collection procedures and intervention fidelity and adherence. The secondary objectives are to derive preliminary estimates of the effect of the intervention on sitting, physical activity and health-related outcomes.

## Methods

### Study design and randomisation

**Fig. 1:**
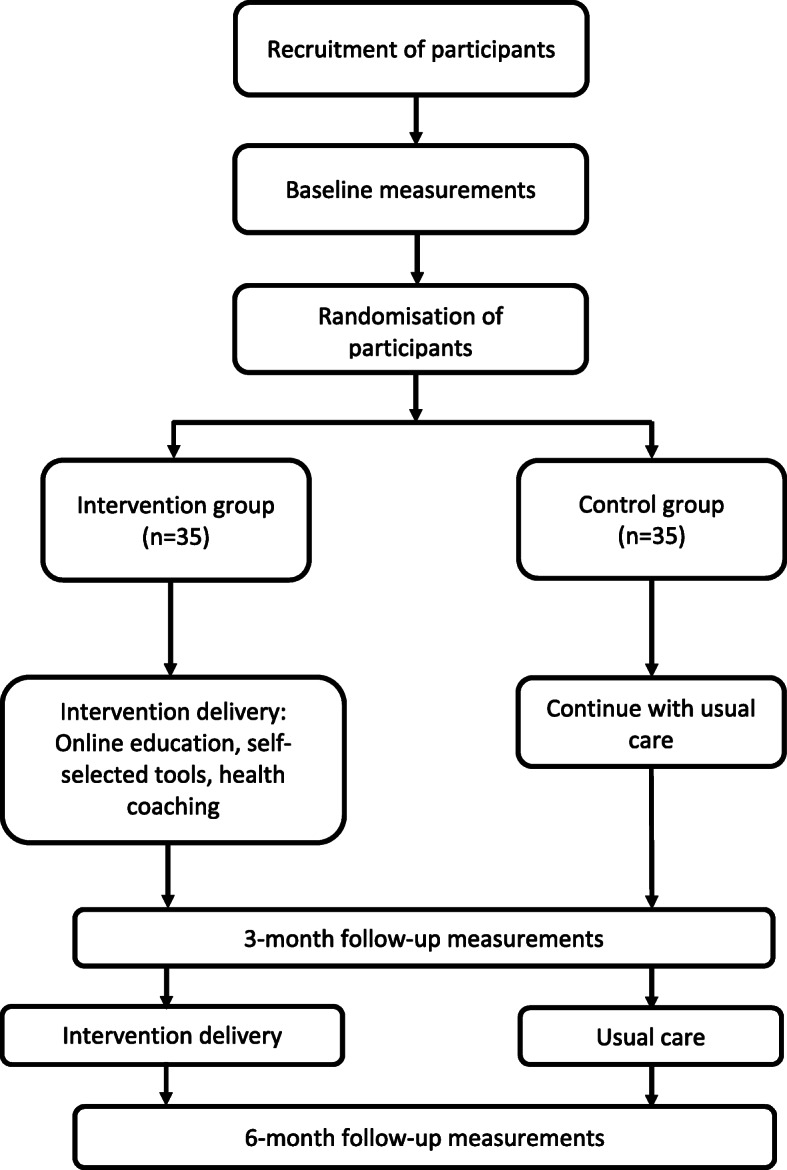
Study design

This will be a mixed-methods randomised controlled feasibility trial taking place in England and conducted, analysed and reported in accordance with the Consolidation Standards of Reporting Trials (CONSORT) statement for pilot and feasibility trials [28]. The study protocol is reported in line with the Standard Protocol Items: Recommendations for Interventional Trials (SPIRIT) statement [29]. After baseline demographic, behavioural- and health-related measures (see below), participants will be individually randomised to either the REgulate your SItting Time (RESIT) intervention or usual care (control group). An independent researcher will use computer generated lists for randomisation of participants in a 1:1 (intervention:control) ratio with a fixed block size of four. The research team and participants will remain blinded to group allocation but, due to the nature of the intervention, the participants and research team members who are in direct contact with them will not be blinded following baseline measures. Further measurements will be conducted at 12 and 24 weeks after baseline measures. The overall study design is shown in Fig. 1. The trial is being conducted during the COVID-19 pandemic. As such, contingency plans are described below, where relevant, with regards to the conduct of the trial if face-to-face research activities are not possible due to restrictions that may be in place because of the pandemic.

### Eligibility criteria

Participants will be eligible if they meet the following inclusion criteria:

- Aged 18-85 years.
- Diagnosed with T2DM. This will be confirmed objectively via a fasting glucose measurement during baseline data collection. *COVID-19 contingency:* If it is not possible to take glucose measures, with consent, each participant’s General Practitioner (GP) will be asked to inform the research team if their patient does not have a T2DM diagnosis.
- Able to ambulate unassisted (with or without the use of a walking aid).
- Self-report sitting for ≥7 h/day. This threshold was selected as mortality risk starts to increase above this volume of daily sitting [30, 31].

Exclusion criteria are as follows:

- Using insulin medication.
- Unable to communicate in English.
- Pregnancy.
- Cognitive or physical conditions interfering with the ability to stand and ambulate.

### Sample size

Sample sizes between 24 and 50 have been recommended for pilot and feasibility studies [32, 33]. A sample size of 70 will be used in this study. This will be sufficient to determine the feasibility of delivering and evaluating the intervention as well as identifying the active ingredients of the intervention in which multiple behaviour change tools can be selected in various combinations by the intervention participants. Furthermore, an *n* of 35 in the intervention group was considered sufficient for exploring participants’ experiences with using different combinations of these tools and whether this was an acceptable intervention approach.

### Participant recruitment

Participant recruitment will be via GP practices where study information will be sent out via postage using Docmail (CFH Docmail Ltd., Radstock, UK) and via GP practice SMS text messaging services to potentially eligible patients identified in a database search. NHS recruitment will be supported by the Clinical Research Network North West London. This is in addition to recruiting via local Diabetes UK support groups (via emails sent out of with a participant information sheet and attendance at group sessions) and social media. A video about the study developed by Health & Care Innovations (Torquay, UK) will be shared on social media and made available on the project webpage (tinyurl.com/RESIT-information). Potentially eligible participants will be asked to express their interest by email, by returning a reply slip to the research team, or by scanning a Quick Response (QR) code provided on recruitment materials that will take them to the project webpage to complete a webform. A researcher will then contact interested volunteers by email/telephone to screen them for eligibility. Individuals who remain potentially eligible will then be invited to complete baseline data collection after providing informed consent.

### The RESIT intervention

The RESIT intervention is intended to last 24 weeks. The intervention was developed following the Medical Research Council guidelines for developing and evaluating complex interventions [34]. An evidence base was identified that supported the need for reducing sedentary behaviour in people with T2DM. Optimal BCTs and their modes of delivery for reducing sedentary behaviour in occupational and non-occupational settings were then identified from previous systematic reviews and published interventions [20, 22, 23, 35–39]. The research team discussed the BCTs and modes of delivery to reach a consensus on those that would be most appropriate for delivering a community-based intervention to reduce sitting in people with T2DM, taking into account factors such as affordability, practicability and acceptability. The BCTs that the RESIT intervention aims to deliver are shown in Table 1. The intervention strategies include an online education programme, health coach support and self-selected behaviour change tools. The use of tailored behaviour change strategies were identified as an efficacious approach for optimising intervention outcomes [24–26]. The strategies through which some BCTs are delivered in this intervention thus vary depending on the behaviour change tools selected by each participant. However, it is intended that each participant will receive the same set of standardised BCTs (Table 1). The RESIT intervention consists of the following strategies.

**Table 1:** Behaviour change techniques [taxonomy label] in the RESIT intervention

Goal setting [1.1]	
Problem solving [1.2]	
Action planning [1.4]	
Review behaviour goals [1.5]	
Discrepancy between current behaviour and goal [1.6]	
Feedback on behaviour [2.2]	
Self-monitoring of behaviour [2.3]	
Social support—unspecified [3.1]	
Information about antecedents [4.2]	
Information about health consequences [5.1]	
Demonstration of the behaviour [6.1]	
Prompts/cues [7.1]	
Credible source [9.1]	
Pros and cons [9.2]	
Social reward [10.4]	

#### Online education programme

After randomisation, all intervention participants will be provided with an online education programme. This is an adapted version of the one used in the SMART Work and Life intervention [40], content of which was based on the face-to-face education used in the SMArT Work multicomponent intervention that led to significant reductions in workplace sitting after 12 months [20]. The online programme includes numerous interactive modules that cover information on the health consequences of excessive sitting, the benefits of reducing sitting, awareness of own sitting time, goal setting and action planning for reducing sitting, addressing potential barriers to sitting less and the importance of self-monitoring and using prompts.

#### Health coach support

Intervention participants will receive support from a qualified health coach. The first consultation will take place 1 to 3 days after completing the online education programme and will be conducted face-to-face or by video call. Subsequent sessions will be conducted by telephone or video call at approximately 2, 6 and 12 weeks after baseline. It is expected that the following BCTs will be used within the health coaching sessions: goal setting, problem solving, action planning, review behaviour goals, discrepancy between current behaviour and goal, feedback on behaviour, self-monitoring of behaviour, social support-unspecified, information about antecedents, information about health consequences, prompts/cues, credible source, pros and cons, social reward and verbal persuasion about capability. The consultations will be semi-structured tailored sessions harnessed on the G.R.O.W (Goal, Reality, Options, Will) model of health coaching [41] that will take each participant through the four stages to enhance intrinsic self-determined motivation, whilst also addressing their capability and opportunity in line with the COM-B (Capability, Opportunity, Motivation – Behaviour) system of behaviour change situated at the hub of the Behaviour Change Wheel [42]. The initial session will focus on a discussion around goals, reality (barriers to reducing sitting using a COM-B behavioural diagnosis), options (self-selected behaviour change tools), and will (confidence to change, how to action plan, monitor and reward progress). Health coaches will use the content of the initial consultation to inform the subsequent coaching sessions, tailored to the participant’s needs and circumstances. These sessions will involve reviewing behavioural goals, problem-solving and progress/adjustment of goals. Participants will be able to contact health coaches via email/telephone/video call outside of planned consultations for additional support if they wish. Each of the health coaches will be trained by the research team to standardise the method of delivery and BCTs used during the intervention.

#### Self-selected behaviour change tools

Participants will be able to select the self-monitoring and prompt tools that they would like to use to support them in reducing and breaking up their sitting time. This includes a suite of commercially available smartphone apps, computer software and wearable devices that assess sitting time/inactive time/computer use and provides feedback and prompts to break up sitting. As described above, the use of tailored behaviour change strategies are more likely to lead to effective outcomes than fully prescribed strategies, as seen when delivering interventions using motivational interviewing [24, 25] and those based on self-determination theory [26]. Our previous intervention work (e.g. the STAND [39], SMArT Work [20], Beat the Seat [22] and MyHealthAvatar-Diabetes [23] interventions) has shown that a ‘one size fits all’ approach is not the most appropriate for achieving reductions in sitting across all participants. Thus, enabling participants to select their own tools for delivery of the same BCT strategy (e.g. self-monitoring of behaviour) may be a more effective approach for reducing sitting.

The self-selected tools will be provided to participants and/or downloaded onto their phone/computer under guidance from a research assistant following completion of the online programme. Participants will be permitted to choose a maximum of one tool from each of the following categories: (a) smartphone app, (b) computer software and (c) wearable device; they are not required to choose a tool from every category if they do not wish to. For example, a participant may choose a smartphone app and a wearable device, but not a computer software tool. The research team will provide a brief guidance document to the participant for each tool selected that will explain how to download it (if relevant) and how it works. Participants will not receive any instructions on how often they should engage with the tools that they select in line with the intervention being person-focused. The chosen behaviour change tools can be changed during the intervention period if a participant reports to the health coach or research team that they are not engaging with the tool. This might be due to reasons such as technical complexity, impracticality or a dislike of the tool. If there are any problems with using any of the tools during the intervention, participants will be able to contact the research team for troubleshooting.

### Control group

Participants randomised to this group will continue to receive their usual diabetes healthcare. They will complete the same measurements (described below) as the intervention group. After the trial has concluded, this group will be offered use of the online education programme and the self-selected behaviour change tools.

### Measurements

After eligibility screening, participants will be invited to complete baseline data collection. This will include the collection of demographic information: date of birth, sex, ethnicity, employment status. The measures below will be collected at baseline and again 12 and 24 weeks later. Information on COVID-19 circumstances will also be collected at each time point including whether participants have had COVID-19 (and date of positive test result if relevant), any changes to employment status (e.g. not working, shift pattern changes, working from home or returning to work) and/or periods of self-isolation. Participants will be required to fast for a minimum of 10 h prior to each data collection session, which will take place at Brunel University London. At the end of this session, participants will be provided with an activPAL4 activity monitor (PAL Technologies, Glasgow, Scotland) to wear for eight days and will return this via post. Each participant will receive a £10 shopping gift voucher for each data collection time point if they take part in the data collection procedures and return the activity monitor to the research team. Data will be entered and managed securely online using Qualtrics (Qualtrics, London, UK). The Qualtrics platform will also be used by participants for completing the study questionnaires.

#### Trial feasibility and intervention acceptability

Trial feasibility will be assessed based on recruitment and retention rates in addition to missing data rates for each of the study measurements. Acceptability and fidelity of the intervention will be assessed as part of the process evaluation described below.

#### Expected main outcomes for a full trial

The expected primary outcome in a definitive full trial is average daily sitting time. The expected secondary outcomes include behavioural (prolonged sitting time, number of breaks in sitting, standing time, and steps), cardiometabolic health, physical function, sleep, musculoskeletal and psychological well-being measures.

##### Sitting, standing and stepping

Sitting, standing and stepping will be measured using the activPAL4 activity monitor worn on the thigh for 24 h/day for eight consecutive days. The activPAL provides valid and reliable assessment of sitting, standing, stepping and postural transitions [43–47]. A diary will be completed by participants for each day that they wear the activPAL to record the time they woke up and got out of bed, the time they went to bed and went to sleep, any times they worked, and any periods when the device was removed [48]. The device will be waterproofed using a nitrile sleeve and medical dressing (Hypafix Hypoallergenic Tape; BSN Medical Limited, Hull, UK) prior to being attached to the right thigh using a Hypafix dressing. Participants will be asked to remove the activPAL when swimming in case it becomes detached and lost. *COVID-19 contingency:* the activPAL activity monitors will be issued and returned exclusively by post.

##### Body composition, anthropometry and blood pressure

Height will be measured using a stadiometer. Weight and body fat % will be measured using the TANITA MC-780MA P segmental body composition analyser (Tanita Corporation, Tokyo, Japan). This device provides a reliable and valid estimate of body fat % using bio-electrical impedance analysis [49]. Participants will wear lightweight clothing and remove shoes and socks for this measurement. Body mass index will be calculated as weight (kg)/height^2^ (cm). Waist circumference will be measured using an adjustable tape measure (Seca 201, Seca Ltd, Birmingham, UK) to the nearest 0.1 cm at the level of the umbilicus at the end of gentle expiration. Blood pressure will be measured on the right arm in a seated position using an Omron M5-I automatic monitoring device (Omron Corporation, Kyoto, Japan). After 5 min of rest, the first reading will be taken followed by two further readings with a 2-min rest between each. The average of the lowest two recordings will be used for analysis. *COVID-19 contingency:* Participants will be asked to measure their waist circumference following standard protocols during a video call with a researcher. They will be provided with a Seca 201 tape measure and receive verbal guidance on how to take the measurement. Body fat %, height and weight measurements will not be taken.

##### Biochemical measures

A finger prick technique will be used to collect fasting capillary blood samples. Glycated haemoglobin will be analysed using the Quo-Test HbA1c analyser (EKF Diagnostics, Cardiff, UK). Fasting total cholesterol, HDL-C, triglycerides and glucose concentrations will be determined using the CardioChek PA point-of-care-system (PTS Diagnostics, Indianapolis, USA). *COVID-19 contingency:* These measures will not be taken if in-person data collection is not possible. In this scenario participants would not be required to fast prior to any measurements being taken.

##### Psychological, sleep, musculoskeletal and well-being measures

Perceived fatigue will be measured using the Chalder Fatigue Scale [50]. Behaviour-specific self-efficacy for reducing sitting will be assessed using an adapted version of the Schwarzer and Renner Physical Exercise Self-Efficacy Scale [51]. General perceived sense of control over one’s actions and outcomes will be assessed using the Generalised Self-Efficacy Scale [52]. The Cohen Perceived Stress Scale will assess perceived stress [53]. To assess the affective aspect of subjective well-being, the Positive and Negative Affect Schedule will measure positive and negative mood [54, 55]. The World Health Organization Five Well-Being Index will measure psychological well-being [56] and quality of life will be measured using the WHOQOL-BREF questionnaire [57]. The Pittsburgh Sleep Quality Index questionnaire will assess sleep quality and duration [58] and musculoskeletal symptoms will be measured using the Standardised Nordic Questionnaire [59].

##### Physical function

Physical function will be assessed using the Short Physical Performance Battery (SPPB) [60] and hand grip strength. The SPPB includes measures of standing balance, walking speed and rising from a chair. Standing balance will be tested using tandem, semi-tandem and side-by-side stands. For each stand type, the researcher will first demonstrate the task and will then support one arm whilst the participant positions their feet. They will then release support when the participant is ready and start timing. The timing will stop when the feet move, the participant grasps the researcher for support, or once 10 s has passed. For the semi-tandem stand, the heel of one foot is placed to the side of the first toe of the other foot. If they are unable to hold this position for 10 s, participants will be assessed with the feet in the side-by-side position. If the semi-tandem position is held for 10 s, participants will also perform the full tandem position with the heel of one foot directly in front of the toes of the other foot. Walking speed will be assessed using an 8-foot walking course with participants instructed to ‘walk to the other end of the course at your usual speed, just as if you were walking down the street to go to the store’. The walk is timed and will be performed twice with the fastest time used for analysis. For the rising from a chair test, participants will be instructed to fold their arms and stand up from a straight-backed chair once. If they perform this successfully, they will then stand up and sit down as quickly as possibly five times whilst being timed. Hand grip strength will be measured on the dominant hand using a digital hand grip dynamometer (Takei Scientific Instruments Co., Ltd, Niigata, Japan) whilst in a standing position with the shoulder adducted and neutrally rotated and the elbow in full extension. Three maximum attempts will be performed with a 1-min rest between each and the average recorded [61]. *COVID-19 contingency:* For the standing balance test, participants will be advised to do this next to a table for support instead of a researcher. For walking speed, participants will be asked to identify an 8-foot clear space where they can perform the test and will be provided with materials to mark out an 8-foot distance. The chair sit to stand task will be performed at home using a chair that can be positioned steady against a wall. The tests will be completed during a video call between the participant and a member of the research team. Grip strength will not be measured.

#### Process evaluation

The process evaluation will include an assessment of the number of participants recruited via the different recruitment strategies and reasons for ineligibility and withdrawal during the trial. Questionnaires and interviews will be used to explore how the intervention was experienced by the participants, the contextual factors that might affect the implementation and outcomes of the intervention, any contamination between the study groups, the implementation fidelity of the trial and intervention and any adaptations that are required to the intervention. Self-report questionnaires will be completed by all participants to explore if the data collection measurements encouraged them to change their behaviour. Intervention participants will additionally answer questions exploring their engagement with the different intervention components. Semi-structured interviews will be completed with a subset of intervention participants to evaluate acceptability of the intervention in terms of compliance with intervention components, facilitators and barriers to participation/compliance and choices made regarding the self-selected behaviour change tools. Interviews will also assess suitability of data collection procedures with a subset of control and intervention participants. Health coaches will be interviewed to assess the feasibility and fidelity of delivering the coaching sessions and will be asked to record 30% of their sessions. These recordings will be used in training booster sessions to monitor GROW and BCT delivery and offer additional support to boost fidelity. The interviews and recorded coaching sessions will be transcribed verbatim. Additional contact points with health coaches outside of planned consultations will also be recorded. Completion rates for the online education programme and health coaching sessions will be collected. The number of times each self-selected behaviour change tool is chosen, the combinations in which they are chosen, any switches in these tools made during the intervention and reasons for the switches will also be recorded and evaluated.

#### Cost-effectiveness

Cost-effectiveness of the intervention is intended to be included in a future full trial. The feasibility of collecting data to inform a cost-effectiveness evaluation will be explored in this study. This will include estimating the cost of the intervention in terms of the required equipment and resources (e.g. cost of wearable devices, phone apps and computer software) in addition to the cost of training and delivery of the health coaching. Quality-adjusted life-years will also be derived from the WHOQOL-BREF measure [57].

### Data analysis

Participant eligibility rates will be calculated as the number of participants eligible / number of participants assessed for eligibility × 100. Recruitment rate will be calculated as the number of participants randomised / number of eligible participants screened × 100. Retention rate for the trial will be calculated as the number of participants who complete follow up measures / number of participants enrolled into the study × 100. Completion rates for the data collection measures will be calculating as the number of complete datasets for each outcome measure / number of participants enrolled into the study × 100.

Interviews will be analysed deductively using Content Thematic Analysis [62], which will be facilitated using the NVivo software (QRS International Pty Ltd, Victoria, Australia). This will enable identification of themes relative to APEASE criteria of the Behaviour Change Wheel [42] to explore Affordability (Can it be delivered to budget?), Practicability (Can it be delivered as designed?), Effectiveness and cost-effectiveness (Does it work, is it cost-effective?), Acceptability (Is it judged appropriate by relevant stakeholders?), Side-effects/safety (Does it have any unwanted side-effects or unintended consequences?), and Equity (Will it reduce or increase the disparities in health/well-being and can it be accessed without causing disparity?).

Each intervention strategy and BCT will be scored for each of the APEASE criteria [63]. Rating scales concerning the intervention will be analysed using descriptive statistics (means, SD, frequencies) and the open-ended responses will be used to identify relevant themes that may explain the quantitative responses provided. The health coaching session recordings will be coded using the list of BCTs included in the trial and the Motivational Interviewing Treatment Integrity scale [64] to assess delivery fidelity. Coders will also listen out for any additional (unintended) BCTs that may have been used that were not in the protocol.

Changes in the descriptive data for each of the study outcome measures will be calculated for the intervention and control groups to explore preliminary efficacy of the intervention. Continuous data will be summarised in terms of mean ± SD. Categorical data will be summarised in terms of frequency, counts and percentages. All baseline variables will be tabulated by the treatment allocation and the overall sample. Descriptive summaries will be generated using SPSS (IBM Corp., Armonk, NY, USA).

### Progression criteria to full trial

The feasibility trial will be deemed successful and lead to a definitive full trial if the following criteria are met:

- The number of required participants is recruited in the planned timeframe for this study.
- ≥70% of participants are retained in the study and provide valid activPAL data at both baseline and subsequent measurement timepoints.
- The qualitative analysis indicates that the participants found the intervention to be acceptable.

If these thresholds are not achieved, barriers to participation and potential adaptations to the study will be explored to identify if issues can be overcome in a full trial.

## Discussion

Adults with T2DM engage in high amounts of sitting [6–8], increasing their risk of hyperglycaemia, CVD and poor psychological well-being [6, 9]. However, there is a paucity of research that has evaluated the feasibility of interventions aimed at reducing and breaking up sitting in ambulatory adults with T2DM. Furthermore, to the authors’ knowledge, there are no studies that have evaluated the feasibility or effectiveness of a tailored intervention aimed at reducing and breaking up sitting in which participants with T2DM are able to select their own behaviour change tools. This study will thus determine the feasibility of conducting an RCT of a tailored intervention to reduce and break up sitting in ambulatory adults with T2DM.

The strengths of the study include the robust randomised controlled trial design to explore the feasibility of conducting an RCT of a tailored intervention to reduce and break up sitting in adults with T2DM. The systematic theory and evidence-driven approach used in the development of the intervention is a further strength. In addition, the embedded process evaluation will identify how the intervention is experienced by the participants and health coaches, the contextual factors that affect implementation and any adaptations that may be needed for a future trial.

The results of the study will enable a decision regarding whether or not a full RCT should be conducted and, if so, will inform its development so that the effectiveness and sustainability of a tailored intervention to reduce and break up sitting in ambulatory adults with T2DM can be determined. This feasibility trial will also provide insight for future studies concerning the promotion of health and well-being in this population group.

## Data Availability

Not applicable.

## Abbreviations

APEASE: Affordability, Practicability, Effectiveness and cost-effectiveness, Acceptability, Side-effects/safety, and Equity
BCT: Behaviour change technique
CVD: Cardiovascular disease
GP: General Practitioner
HDL-C: High-density lipoprotein cholesterol
NHS: National Health Service
RCT: Randomised controlled trial
RESIT: REgulate your SItting Time
SPPB: Short Physical Performance Battery
T2DM: Type 2 diabetes mellitus
